# Quantitative Analysis of the Human Face Skin Thickness—A High-Frequency Ultrasound Study

**DOI:** 10.3390/jcm14238401

**Published:** 2025-11-27

**Authors:** Szymon Korzekwa, Krystian Matusz, Michał Kukulski, Paweł Wawrzaszek, Natalie Górna, Włodzimierz Rosiński, Jakub Włosiański, Agnieszka Przystańska

**Affiliations:** 1 Department of Anatomy, Poznań University of Medical Sciences, 61-701 Poznan, Poland; 2 QKA Esthetic Medicine Poznań, 61-553 Poznan, Poland; 3 Department of Orthodontics and Temporomandibular Disorders, Poznań University of Medical Sciences, 61-701 Poznan, Poland; 4 Aestethic Clinic Poznań, 60-142 Poznan, Poland

**Keywords:** high-frequency ultrasound, facial skin thickness, esthetic surgery

## Abstract

Understanding the topography of facial skin thickness is crucial in plastic surgery and underpins the success of numerous reconstructive, oncological, transplant, volumetric, and aesthetic procedures. **Objective**: This study aims to assess facial skin thickness using high-frequency ultrasound, evaluate its correlation with age, anatomical location, and physical parameters, and create a comprehensive facial skin thickness map. **Methods**: High-frequency ultrasound (75 MHz, axial resolution 21 μm) was used to scan 38 anatomical sites on the faces of 45 patients aged 22–73 years from esthetic medicine clinics. Measurements were performed manually within the device software. Extensive statistical analysis was conducted to determine dominant thickness values, assess variations by location and age, and evaluate correlations between biometric variables (age, weight, height, BMI) and skin thickness at various facial sites. Based on these findings, a relative thickness index and detailed topographic map of facial skin were developed. **Results**: The thickest skin was observed in the lower third of the nose, particularly at the nasal apex (median: 1907 μm, IQR: 455.75 μm, *p* < 0.001), while the thinnest skin was on the upper eyelid (median: 573.50 μm, IQR: 128.75 μm, *p* < 0.001). Skin thickness was significantly influenced by anatomical location (*p* < 0.001, η^2^ = 0.52), age (*p* < 0.001, η^2^ < 0.01), and their interaction (*p* < 0.001, η^2^ = 0.04), collectively accounting for approximately 57% of variance in skin thickness. Correlations between biometric parameters and skin thickness were generally weak, though a few strong correlations were found between specific facial sites. **Conclusions**: A detailed facial skin thickness map, including a relative thickness index, was developed.

## 1. Introduction

The thickness of facial skin is a critical factor in several medical fields, particularly plastic surgery and esthetic medicine. As early as 1951, Barker investigated the distribution of skin thickness across the human body in effort to improve grafting outcomes. His micrometric measurements, while groundbreaking, were conducted on limited cadavering samples, highlighting the need for an in vivo assessment to obtain more accurate data [1].

Since then, a growing body of research has emphasized the clinical importance of understanding skin thickness. Taylor, for instance, demonstrates its significance in nasal correction procedures, where skin thickness influences both surgical feasibility and postoperative outcomes, including periorbital bruising and edema [2,3]. Furthermore, accurate assessment is necessary for selecting and harvesting appropriately thick skin grafts [4], planning resections and reconstructions [5], oncological procedures [6], wound and scar management [7], and in the administration of injectable neuromodulators [8,9,10] or soft tissue fillers used in esthetic interventions [11].

In esthetic procedures such knowledge is especially valuable, since most interventions target the dermis or the dermal–subcutaneous junction, using both synthetic and autologous fillers [12,13]. Yet, despite its relevance, comprehensive studies on regional skin thickness in commonly treated facial zones remain limited and the topic is often overlooked [14]. Where such data do exist, they are primarily applied in rhytidectomy (facelift) planning [15,16].

While it is common knowledge that the skin of the eyelid is thin and that of the nose is thick, such descriptions lack quantitative precision [17]. In fact, some sources do not differentiate skin thickness across the face at all [1]. For practitioners performing facial procedures, detailed and reliable knowledge of regional skin thickness is essential, yet literature addressing this need remains surprisingly sparse.

To date, only a few researchers have investigated skin thickness, and most have focused on specific diseases [18,19], treatment outcomes [20], harmful exposures [21], or forensic applications [22,23]. Most studies examine the total soft tissue thickness of the face for reconstructive purposes [24,25], commonly using imaging techniques such as computed tomography (CT). However, CT lacks sufficient resolution to differentiate individual soft tissue layers [26,27,28].

Studies dedicated exclusively to skin thickness are rare and often methodologically limited. For example, Dykes et al. (1976) used calipers and radiographic images to estimate skin and subcutaneous fat thickness—methods prone to significant measurement error [29]. Among the few studies in the context of plastic surgery, Ha et al. [17] and Chopra et al. [30] have used optical microscopy of biopsied tissue, but their work was hindered by small sample sizes. Ha et al. examined only three unpreserved cadavers across 15 facial sites, while Chopra et al. expanded to 24 sites but still relied on just 10 cadavers. Both studies were further limited by skewed sex distribution (only 30% male) and advanced subject age (mean ages 70 and 81.6 years) which undermines statistical validity and precludes age-related analysis. Moreover, the accuracy of postmortem biopsy is compromised by early tissue dehydration, which distorts skin thickness [22].

High-frequency ultrasonography (HFUS) has been proposed as a non-invasive alternative and is widely regarded as the gold standard for in vivo skin thickness assessment. This method offers excellent tissue visualization, enabling precise delineation of the skin–subcutaneous interface. In dermatologic applications, its resolution is approximately 30–60 times smaller than typical skin thicknesses, which allows for accurate measurements of individual layers. Reported depth measurement errors with HFUS are as low as 3% [31]. While higher-frequency probes improve resolution at the expense of penetration depth [32], HFUS remains a highly sensitive, non-invasive, reliable, and reproducible tool. It is already well established in skin cancer diagnostics for its quantitative, reproducible, and responsive characteristics [33] and holds significant promise for broader applications in plastic surgery and esthetic medicine.

The aim of this study was to analyze skin thickness in selected regions of the human face using non-invasive imaging with high-frequency ultrasonography (HFUS). The goal was to validate the effectiveness of this method in a relatively large population of female patients, to assess correlations between measured parameters, and to develop a comprehensive facial skin thickness map.

The working hypothesis was that the apex of the nose would have the greatest skin thickness, whereas the upper eyelid would exhibit the thinnest skin layer [30,34].

## 2. Materials and Methods

This study is based on a retrospective analysis of 45 female patients who attended an esthetic medicine clinic. The original design aimed to include 15 patients in each of three age groups: 20–35, 36–50, and over 51 years old. However, after reviewing the age distribution and applying quartile segmentation, the final age groups were defined as 0–33, 33–40, 40–54, and 54–73 years old.

All measurements were performed prior to any esthetic procedures. Patients with visible or physician-diagnosed skin conditions were excluded, as well as those with prior esthetic procedures in the examined facial areas, confirmed either through medical history or HFUS imaging.

**Figure 1:**
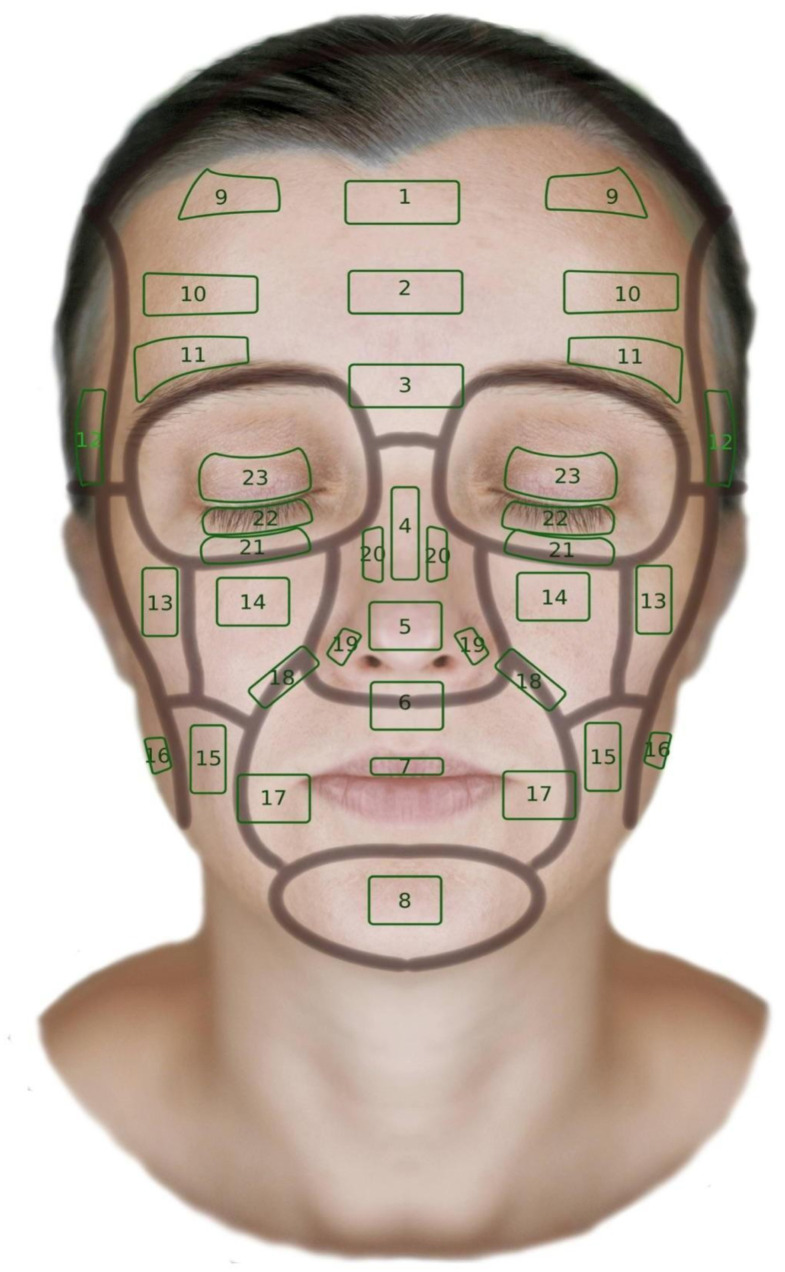
Examined areas mapped onto a facial diagram. 1—upper forehead region; 2—forehead region; 3—glabella; 4—nasal dorsum; 5—nasal tip; 6—philtrum; 7—upper vermilion lip; 8—mental region; 9—superolateral forehead region (left/right); 10—lateral forehead (right/left); 11—supraorbital region (right/left); 12—temporal region (right/left); 13—zygomatic region (right/left); 14—infraorbital region (right/left); 15—buccal region (right/left); 16—mandibular angle (right/left); 17—oral commissure or lower nasolabial fold (right/left); 18—nasolabial fold (right/left); 19—nasal ala (right/left); 20—lateral nasal surface (right/left); 21—tear trough (right/left); 22—lower eyelid (right/left); 23—upper eyelid (right/left).

For the purposes of this study, 23 distinct facial regions were selected, of which 15 were bilaterally symmetrical (left/right) lateral areas, resulting in a total of 38 anatomical sites assessed per patient. High-frequency ultrasound examinations were performed in the following regions for each participant (Figure 1).

**Figure 2:**
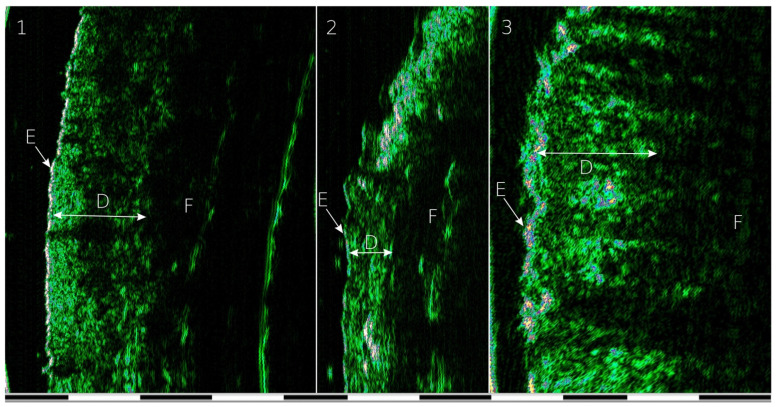
Examples of HFUS images: 1—cross-section of the skin of the forehead region, 2—cross-section of the skin of the upper eyelid, 3—cross-section of the skin of the nasal tip, E—epidermis, D—dermis, F—subcutaneous fat. The image scale is shown in the lower bar, where one section is 1 mm.

Skin thickness images were acquired using a high-frequency ultrasound device, the DUB SkinScanner75 (software version 3.14, TPM GmbH, Lüneburg, Germany), equipped with a mechanical 75 MHz linear probe (axial resolution: 21 μm, lateral resolution: 33 μm, scanning depth: 3.2 mm). Examples of images of the forehead skin, upper eyelid and tip of the nose are shown in Figure 2.

Measurements were performed manually using the integrated measurement tools within the DUB SkinScanner software (TPM GmbH, Lüneburg, Germany). For each cross-sectional B-scan image, ten measurements were taken at 1 mm intervals, with supplementary reference to A-scan projections. In total, more than 8000 individual measurements were recorded.

All measurements were conducted parallel to the axis of the ultrasound beam. The Rectangle Measurement tool was used to minimize angular aberration, and the recorded parameter was Depth.

For each image, selected based on the perpendicular alignment of the tissue surface to the ultrasound beam axis, ten measurements were taken at 1 mm intervals along the vertical axis (perpendicular to the beam axis). The measurement lines were guided using micro-positioners to eliminate discrepancies caused by potential hand movement or vibration.

### Statistical Analysis

To address the researchers’ questions, statistical analyses were performed using the R programming language within the RStudio environment (ver. R 4.1.0). The analysis employed several packages, including ggplot2 and corrplot for data visualization, rstatix for hypothesis testing, and lmPerm and rcompanion for permutation tests.

The primary hypothesis tested was whether skin thickness varied significantly depending on anatomical location. Normality within groups was assessed using skewness and kurtosis coefficients, supplemented by the Anderson–Darling test. Group sizes were also assessed for equality. Due to considerable deviation from normality and unequal group sizes, the non-parametric Kruskal–Wallis test was employed as an alternative to one-way ANOVA. Post hoc analyses were performed using Dunn’s test with Holm’s correction for multiple comparisons, and effect sizes were reported as η^2^.

Subsequently, a two-factor permutation model (23 × 4 design) was constructed to evaluate the interaction between anatomical location and age. Post hoc testing was carried out using permutation tests of independence with Benjamini–Hochberg (1995) [35] correction, with η^2^ again serving as the effect size measure.

Finally, correlation analysis between continuous variables was conducted. First, associations were examined between central location and age, weight, height, and BMI, followed by lateral location with the same biometric variables. A final correlation matrix comprising all 27 variables (27 × 27) was generated. The global significance level for all statistical comparisons was set at α = 0.05.

## 3. Results

### 3.1. Differences in Skin Thickness Depending on Anatomical Location

**Graph 1:**
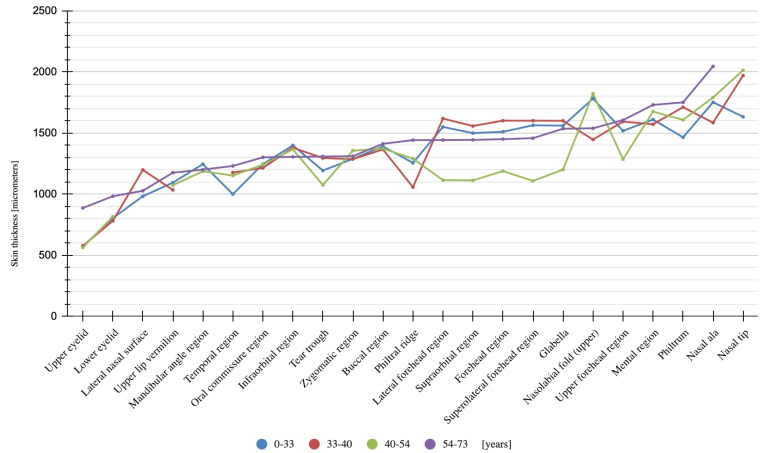
Line chart illustrating skin thickness by location for each age group.

**Figure 3:**
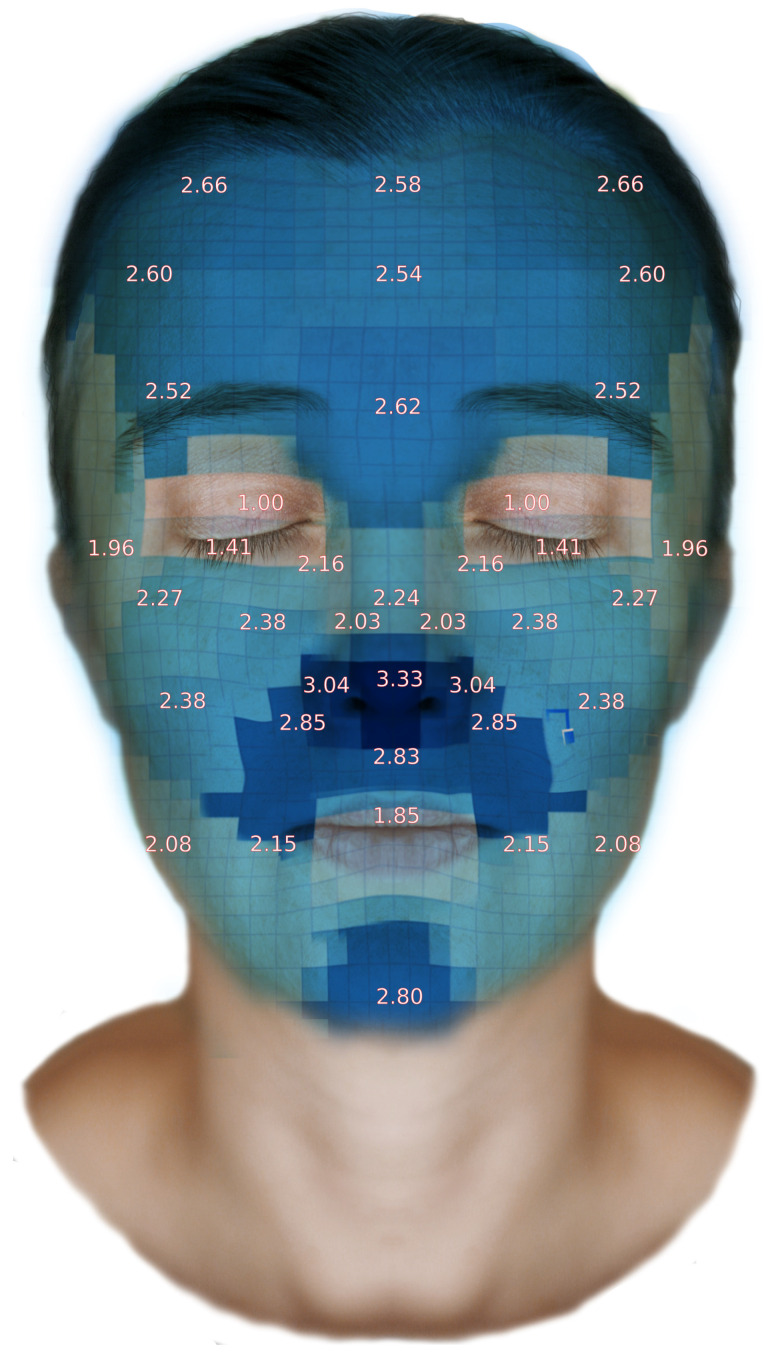
Matching the facial skin thickness map to an anatomical model. RTI values were entered directly at their corresponding location. Facial skin thickness in mm.

To test the hypothesis regarding the presence of differences in facial skin thickness depending on anatomical location, a Kruskal–Wallis test was performed, followed by multiple pairwise comparisons using Dunn’s test with Holm’s correction. Due to the high number of post hoc comparisons (253) and many statistically significant differences (200), the results were additionally visualized using an arc diagram generated with the ggraph package. Post hoc test results are also presented in tabular form. The findings are summarized in Table 1 and Table 2, Graph 1 and Figure 3.

### 3.2. Differences in Skin Thickness by Anatomical Location

The results of the Kruskal–Wallis test pointed to statistically significant differences in skin thickness depending on anatomical location. Based on the η^2^ coefficient, these differences appear substantial: the between-subject factor (location) accounts for approximately 50% of the variance in the dependent variable (skin thickness). Post hoc tests further confirmed the presence of many significant pairwise differences. Only a few comparisons shown in Graph 1 were found to be non-significant.

### 3.3. Variation in Skin Thickness by Location and Age

To assess the variation in skin thickness with respect to both anatomical location and age, a 23 × 4 permutation-based ANOVA was conducted, including the interaction effect between the two factors. Age groups were defined based on the distribution of the variable and quartile segmentation. Median values, interquartile ranges, and sample sizes for each subgroup are presented in Table 3.

### 3.4. Facial Skin Thickness Map

Based on the obtained measurement results, a facial skin thickness map was developed. A spatial coordinate system was created using Numbers spreadsheet software (ver. 11.1), and three-dimensional interpolation was performed in Matlab to estimate values in areas not directly covered by measurements. Subsequently, the Relative Thickness Index (RTI) was calculated and normalized to the site with the lowest measured skin thickness [30]. It is presented as Figure 3.

### 3.5. Analysis of Skin Thickness Depending on Facial Location

The fundamental hypothesis, that skin thickness varies across different facial regions, was confirmed beyond any doubt. The large number of measurements used for statistical calculations demonstrated that anatomical location alone accounts for approximately 50% of the variance in skin thickness. No other factor exerted a comparable influence.

As expected, the thinnest skin was found on the upper eyelid, while the thickest was observed at the tip of the nose.

Analyzing the distribution of skin thickness values reveals that the greatest thickness is observed in the most anteriorly projecting areas of the face: the nasal, mental (chin), and frontal regions.

Overall, the thickest skin tended to occur in the most anteriorly projecting areas of the face (nasal, mental, and frontal regions), whereas the thinnest skin was found in recessed or more protected regions, such as the periorbital and temporal areas.

More specifically, the thinnest skin was found on the upper eyelid, followed by the lower eyelid, then the lateral surface of the nose, the vermilion of the upper lip, and the mandibular angle, temporal region, and oral commissure. Slightly thicker, yet comparable skin was found in the infraorbital area (tear trough), the zygomatic and the buccal region. This was followed by the nasal dorsum and the entire forehead (including the lateral forehead, supraorbital area, central forehead, superolateral forehead, and glabella). The thickest skin was measured in the perioral region, close to the nose (nasolabial fold, philtrum), the mental region, and—with the highest recorded values—on the nasal ala and the nasal tip.

These findings were compared with the limited existing literature. To date, only two studies provide a comprehensive assessment of facial skin thickness: Ha et al. [17] and Chopra et al. [30], the latter representing, in the authors’ view, an extension of the former’s work [17,30].

**Graph 2:**
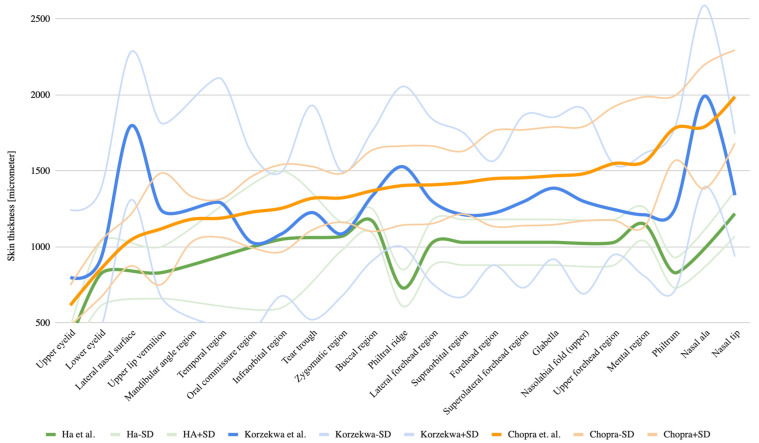
Comparison of literature data (Ha et al. [17] and Chopra et al. [30]) with the results obtained in the present study. Lines with lower color saturation above and below each main curve represent the standard deviation for each study.

Graph 2 presents a comparative graph of all three datasets: the green line corresponds to Ha et al. [17], the blue line to Chopra et al. [30], and the orange line to the present study, with adjacent curves illustrating standard deviation ranges.

Graph 2 clearly demonstrates that the results of Ha et al. [17] and Chopra et al. [30] do not align with each other. Ha et al.’s [17] data, in almost all facial regions, correlate closely with the results of the present study, except for two areas: the nasal dorsum (730 μm) and the philtrum (830 μm), where Ha’s values are significantly lower. Aside from these exceptions, the trend lines are similar, though Ha’s results are consistently about 30% lower on average. This discrepancy could potentially be attributed to a systematic factor such as measurement bias or methodological limitations such as tissue shrinkage due to biopsy preparation.

In contrast, the results of Chopra et al. [30] show considerably greater variability. Differences range from −30% in the upper eyelid region to +30% in the philtrum with the lateral nasal wall differing by more than 70%. However, the authors did not precisely define the puncture site within this region. The highly variable thickness across the nose, with values decreasing sharply toward the glabella, may reflect measurements taken in anatomically distinct locations, which could explain the observed discrepancies. However, a more in-depth analysis, considering the overall measurement dispersion (hence the use of an atypical chart that includes ±2σ boundaries), indicates that the HFUS measurements obtained in this study largely fall within the 2σ range reported by Chopra et al. [30]. Furthermore, we attribute these discrepancies to significant methodological and statistical differences in the studies conducted by both authors. Ha conducted measurements on three cadaver specimens, from which he collected biopsies and performed histometric measurements on those biopsies, preserved in formalin, sectioned, and stained with hematoxylin and eosin. Chopra, on the other hand, performed measurements on 10 cadaver specimens using the full-thickness punch biopsy method and fixing the tissue specimens in paraffin-embedded slides. This paper presents in vivo measurements.

### 3.6. Analysis of Skin Thickness by Age Group

Skin thickness patterns do not follow the same sequence across all age groups. Moreover, not all age groups confirm the previously mentioned finding that the thickest skin is located at the nasal tip. In fact, in the youngest age group, the nasal tip was relatively thinner, with the nasal ala emerging as the thickest site.

Extending the analysis further, both anatomical location and patient age, as well as the interaction between these variables, significantly influenced facial skin thickness. However, while location explains a substantial portion of the variance, age has only a modest effect, which was an unexpected outcome.

Contrary to the common assumption that skin becomes progressively thinner with age our findings did not confirm this phenomenon. Taken together, location and age explained approximately 57% of the variance in skin thickness.

Notably, in the oldest age group (54–73 years), there was a marked increase in skin thickness in the nasal area (nasal tip, nasal ala, and philtrum), consistent with literature indicating a positive correlation between age and skin thickness in the nasal region (rs = 0.407, *p* = 0.000) [34]. This challenges the prevailing view that age-related thinning of nasal skin unmasks dorsal or tip irregularities previously concealed by greater thickness in youth.

A similar pattern was observed in the forehead region—but only when comparing the oldest group to the immediately younger group (40–54 years). The forehead is of particular interest in this study, as it was subdivided into six distinct measurement subregions. In the younger age groups (0–33 and 33–40 years), skin thickness remained similar and followed a consistent trend. However, in the 40–54 age group, a sharp decline of nearly 500 μm was observed, followed by a return to previous levels in the 54–73 age group. This abrupt change is puzzling and difficult to explain. If it were due to measurement error, the sample size should have mitigated its effect. Furthermore, the fact that the entire anatomical area was affected argues against measurement artifacts.

To clearly illustrate additional relationships, a skin thickness map was generated based on averaged results. This map provides an excellent visual representation of the overall skin thickness distribution and may serve as a valuable reference for procedures requiring knowledge of skin depth. Additionally, the map includes values for the Relative Thickness Index (RTI), normalized to the thinnest region, as recommended in the literature [17].

### 3.7. Correlation Analysis of Quantitative Variables: Age, Weight, Height, and BMI

Correlation matrices revealed strong associations for only a few measured parameters. As expected, a strong correlation was found between weight and BMI (r = 0.92), as well as between age and weight (r = 0.60). None of the anatomical regions demonstrated a significant correlation with BMI, consistent with previous reports [34]. Similarly, correlations between anatomical regions and age were not found.

### 3.8. Correlation Between Skin Thickness in Different Anatomical Regions

Significant correlations between anatomical locations were limited. Strong relationships were found within the forehead region, particularly between the upper frontal, central frontal, and glabellar areas (r = 0.82–0.85). Other regions showed weaker or even inverse correlations, such as between the frontal region and both the lateral nose (−0.51) and the chin (−0.47).

In the lateral face, correlations were found between the upper lateral frontal, lateral frontal, and supraorbital areas (r = 0.82–0.85). Moderate correlations were present in the infraorbital, buccal, zygomatic, and mandibular angle regions (r = 0.53–0.63). The mandibular angle also correlated with the nasolabial fold (r = 0.86). The lateral nose correlated with the nasal ala (r = 0.70) and the zygomatic region (r = 0.58). Additionally, the lateral and upper-lateral forehead and the supraorbital region showed moderate correlation with the tear trough area (r = 0.50–0.64).

A negative correlation was observed between the lateral nasal surface and the upper eyelid (r = −0.62).

Many authors have stated that ultrasonography, particularly high-frequency ultrasound (HFUS), provides excellent visualization of skin and subcutaneous tissue structures. Numerous studies have validated HFUS as a reliable method for imaging microanatomical structures [36,37]. Although HFUS is not yet fully standardized, some researchers have developed protocols for selected anatomical regions [38].

HFUS shows good agreement with histological measurements (ICC = 0.807; 95% CI: 0.703–0.877) and excellent interobserver reproducibility (G = 0.97), supporting its reliability in the in vivo assessment of melanoma thickness. In contrast, optical coherence tomography showed poor agreement with histopathological analysis (ICC = 0.0; 95% CI: −0.2 to 0.2) and no interobserver consistency (G = 0.00) [39]. Other authors confirm the use of HFUS in oncology, indicating that it is a reliable, reproducible, and non-invasive method for assessing skin lesion thickness (e.g., melanoma) [40,41,42]. Routine use of HFUS may allow for single-stage excision of melanocytic lesions with surgical margins determined by in vivo tumor thickness measurements.

Ultrasound has also been used to guide the depth of botulinum toxin type A (BoNT-A) injections. Studies confirmed that clinicians can use ultrasonography to identify the structural layers of the forehead and predict soft tissue thickness to optimize injection depth [10].

## 4. Discussion

From a clinical perspective, the measurement of skin thickness provides an objective and reproducible means of monitoring diseases characterized by fibrosis, atrophy, or inflammation. As such, the assessment of skin thickness represents a clinically and scientifically significant component in the evaluation of both dermatologic and systemic disorders. HFUS has emerged as a valuable non-invasive diagnostic tool for skin evaluation, particularly in systemic sclerosis, where progressive dermal thickening is one of the most prominent and extensively studied manifestations [43]. Beyond systemic disease, HFUS-derived measurements of skin thickness and echotexture can serve as dynamic indicators of local tissue health in intraoperative and postoperative settings [44].

In esthetic and reconstructive medicine, the evaluation of skin thickness holds both diagnostic and procedural importance. It informs precise treatment planning for interventions such as dermal filler injections, fat grafting, microneedling, and laser therapy—procedures where detailed knowledge of dermal and subcutaneous dimensions is critical for safety and efficacy. Moreover, skin thickness assessment contributes to understanding age-related structural alterations and supports the development of rejuvenation strategies. Quantitative data on dermal and hypodermal layers also facilitate the objective evaluation of outcomes following energy-based treatments (e.g., radiofrequency or ultrasound skin tightening), where remodeling of collagen and neocollagenesis are reflected in measurable changes in tissue architecture [45].

A noticeable and surprising decrease in forehead thickness appeared in the 40–54 age group, which might suggest (but does not confirm) some form of region-specific acceleration of skin aging during midlife. It is plausible that the frontal dermis could be more vulnerable to collagen loss and elastin fragmentation, and the area’s relatively uniform and thin subcutaneous layer may mean that even modest dermal atrophy could translate into a detectable reduction in total skin thickness [46].

In oncologic dermatology, HFUS plays a complementary role in the evaluation of skin lesion architecture and invasion depth. Increased skin thickness and loss of normal layer delineation can indicate malignant infiltration, while distinct hypoechoic patterns may suggest basal cell carcinoma (BCC) [47].

Overall, quantitative imaging of skin thickness—particularly through high- and ultra-high-frequency ultrasound—offers a non-invasive approach to capturing in vivo structural and functional dynamics of the skin. The integration of these quantitative imaging data into computational and artificial intelligence (AI)-based frameworks further enables the development of predictive models for disease detection, classification, and outcome assessment, marking a significant advancement in personalized dermatologic care.

## 5. Strengths and Limitations

This study stands out in the literature due to its large sample size and broad age distribution of female participants. The age groups were evenly distributed, minimizing potential biases in measurements and subsequent statistical analysis. The reliability of the results is supported by the inclusion of only healthy individuals without dermatological conditions. However, we must acknowledge a potential limitation: participants were all patients from esthetic medicine clinics, possibly introducing outliers due to prior procedures, dermal fillers, or foreign bodies in the skin.

To reduce this risk, patients with a history of major surgical or esthetic interventions involving foreign substances in the skin were excluded. Also excluded were scans with clear evidence of prior interventions.

At the same time, the use of esthetic medicine treatments may indicate greater overall skin-care diligence, which could include cosmetic procedures or routines influencing facial skin condition but remaining undetected during participant qualification. Larger, dedicated studies with more detailed assessments would help minimize this potential source of bias.

Additionally, because all participants were women, the findings cannot be directly generalized to male skin thickness. Future research involving more diverse populations is recommended.

From a statistical standpoint, the even age distribution and large number of scans greatly strengthened the study’s credibility. The measurement methodology yielded a vast number of data points, allowing for robust statistical calculations.

Due to the retrospective nature of the study and the scanning methodology, only one side of each face was analyzed. As a result, facial symmetry was assumed. This raises an interesting question for future research—examining facial asymmetry could provide valuable insights, though this would require a prospective study design. Retrospective studies limit opportunities for repeated or optimized measurements and often suffer from ambiguous or unverifiable markings.

## 6. Conclusions

The results confirm the hypothesis and further specify that, at any age, the thickest facial skin is in the lower third of the nose (particularly at the nasal tip and ala), while the thinnest skin is found on the upper eyelid.

There were no strong interregional correlations between skin thickness measurements, nor there were any strong correlations between these measurements and age, weight, height, or BMI. The primary age-related trend was an increase in nasal tip skin thickness with advancing age.

## Figures and Tables

**Table 1:** Variation in facial skin thickness depending on location.

Side	Area	Sample Size	Median	Interquartile Range
bilaterally symmetrical	tear trough	390	1241.5	343
	upper nasolabial fold	390	1636.5	579
	nasal ala	160	1744	223
	lateral forehead	350	1492	528.75
	superolateral forehead region	360	1523	505.75
	zygomatic region	418	1303.5	199.75
	oral commissure	390	1234	240.75
	mandibular angle	126	1195.5	220.25
	infraorbital region	350	1445	509
	supraorbital region	440	1362.5	298.5
	buccal region	390	1367.5	373.5
	temporal region	390	1126.5	251.75
	lower eyelid	430	807.5	260.75
	upper eyelid	430	573.5	128.75
	lateral nasal surface	50	1163	371
central	philtrum	388	1624	403.25
	nasal tip	430	1907	455.75
	upper vermilion lip	390	1061	270.25
	glabella	360	1503.5	522.75
	nasal dorsum	118	1282	259.75
	mental region	430	1606	560.25
	upper forehead region	450	1480	432.25
	forehead region	360	1456.5	440.25

**Table 2:** Results of Dunn’s post hoc test with Holm correction for facial skin thickness depending on location.

Group 1	Group 2	Sample Size Group 1	Sample Size Group 2	Dunn’s Test Statistic	*p* Value
tear trough (L)	Nasolabial fold (L)	390	390	14.38	<0.001
	Upper lip vermilion (C)	390	390	−5.89	<0.001
	Glabella (C)	390	360	8.93	<0.001
	Mental region (C)	390	430	13.03	<0.001
	Lateral forehead (L)	390	350	9.09	<0.001
	Upper forehead region (C)	390	450	9.6	<0.001
	Superolateral forehead region (L)	390	360	9.39	<0.001
	Frontal region (C)	390	360	8.81	<0.001
	Zygomatic region (L)	390	418	3.87	0.007
	Supraorbital region (L)	390	350	8.01	<0.001
	Infraorbital region (L)	390	440	6.03	<0.001
	Buccal region (L)	390	390	6.5	<0.001
	Temporal region (L)	390	390	−3.73	0.012
	Lower eyelid (L)	390	430	−13.9	<0.001
	Upper eyelid (L)	390	430	−17.61	<0.001
	Philtrum (C)	390	388	15.71	<0.001
	Nasal ala (L)	390	160	15.5	<0.001
	Nasal tip (C)	390	430	22.85	<0.001
Philtrum (L)	Upper lip vermilion (C)	390	390	−20.27	<0.001
	Glabella (C)	390	360	−5.16	<0.001
	Nasal dorsum (C)	390	118	−7.94	<0.001
	Lateral forehead (L)	390	350	−4.89	<0.001
	Upper forehead region (C)	390	450	−5.28	<0.001
	Superolateral forehead region (L)	390	360	−4.7	<0.001
	Frontal region (C)	390	360	−5.28	<0.001
	Zygomatic region (L)	390	418	−10.76	<0.001
	Oral commissure region (L)	390	390	−13.42	<0.001
	Mandibular angle region (L)	390	126	−11.23	<0.001
	Supraorbital region (L)	390	350	−5.97	<0.001
	Infraorbital region (L)	390	440	−8.78	<0.001
	Buccal region (L)	390	390	−7.88	<0.001
	Temporal region (L)	390	390	−18.11	<0.001
	Lower eyelid (L)	390	430	−28.63	<0.001
	Upper eyelid (L)	390	430	−32.34	<0.001
	Lateral nasal surface (L)	390	50	−7.17	<0.001
	Nasal ala (L)	390	160	4.53	<0.001
	Nasal tip (C)	390	430	8.13	<0.001
Upper lip vermilion (C)	Glabella (C)	390	360	14.7	<0.001
	Nasal dorsum (C)	390	118	5.87	<0.001
	Mental region (C)	390	430	19.07	<0.001
	Lateral forehead (L)	390	350	14.82	<0.001
	Upper forehead region (C)	390	450	15.7	<0.001
	Superolateral forehead region (L)	390	360	15.16	<0.001
	Frontal region (C)	390	360	14.58	<0.001
	Zygomatic region (L)	390	418	9.86	<0.001
	Oral commissure region (L)	390	390	6.85	<0.001
	Supraorbital region (L)	390	350	13.74	<0.001
	Infraorbital region (L)	390	440	12.1	<0.001
	Buccal region (L)	390	390	12.39	<0.001
	Lower eyelid (L)	390	430	−7.87	<0.001
	Upper eyelid (L)	390	430	−11.58	<0.001
	Philtrum (C)	390	388	21.59	<0.001
	Nasal ala (L)	390	160	19.99	<0.001
	Nasal tip (C)	390	430	28.88	<0.001
Glabella (C)	Nasal dorsum (C)	360	118	−4.31	0.001
	Mental region (C)	360	430	3.62	0.017
	Zygomatic region (L)	360	418	−5.29	<0.001
	Oral commissure region (L)	360	390	−7.99	<0.001
	Mandibular angle region (L)	360	126	−7.48	<0.001
	Temporal region (L)	360	390	−12.59	<0.001
	Lower eyelid (L)	360	430	−22.74	<0.001
	Upper eyelid (L)	360	430	−26.38	<0.001
	Lateral nasal surface (L)	360	50	−4.64	<0.001
	Philtrum (C)	360	388	6.47	<0.001
	Nasal ala (L)	360	160	8.44	<0.001
	Nasal tip (C)	360	430	13.23	<0.001
Nasal dorsum (C)	Mental region (C)	118	430	6.89	<0.001
	Lateral forehead (L)	118	350	4.46	0.001
	Upper forehead region (C)	118	450	4.54	<0.001
	Superolateral forehead region (L)	118	360	4.63	<0.001
	Frontal region (C)	118	360	4.23	0.002
	Supraorbital region (L)	118	350	3.71	0.013
	Temporal region (L)	118	390	−4.4	0.001
	Lower eyelid (L)	118	430	−11.23	<0.001
	Upper eyelid (L)	118	430	−13.73	<0.001
	Philtrum (C)	118	388	8.86	<0.001
	Nasal ala (L)	118	160	10.38	<0.001
	Nasal tip (C)	118	430	13.5	<0.001
Mental region (C)	Lateral forehead (L)	430	350	−3.36	0.042
	Upper forehead region (C)	430	450	−3.67	0.015
	Frontal region (C)	430	360	−3.74	0.011
	Zygomatic region (L)	430	418	−9.3	<0.001
	Oral commissure region (L)	430	390	−12.06	<0.001
	Mandibular angle region (L)	430	126	−10.2	<0.001
	Supraorbital region (L)	430	350	−4.47	0.001
	Infraorbital region (L)	430	440	−7.26	<0.001
	Buccal region (L)	430	390	−6.38	<0.001
	Temporal region (L)	430	390	−16.86	<0.001
	Lower eyelid (L)	430	430	−27.62	<0.001
	Upper eyelid (L)	430	430	−31.42	<0.001
	Lateral nasal surface (L)	430	50	−6.42	<0.001
	Nasal ala (L)	430	160	5.87	<0.001
	Nasal tip (C)	430	430	10.07	<0.001
Frontal region (lateral) (L)	Zygomatic region (L)	350	418	−5.48	<0.001
	Oral commissure region (L)	350	390	−8.16	<0.001
	Mandibular angle region (L)	350	126	−7.61	<0.001
	Infraorbital region (L)	350	440	−3.49	0.027
	Temporal region (L)	350	390	−12.73	<0.001
	Lower eyelid (L)	350	430	−22.8	<0.001
	Upper eyelid (L)	350	430	−26.41	<0.001
	Lateral nasal surface (L)	350	50	−4.74	<0.001
	Philtrum (C)	350	388	6.2	<0.001
	Nasal ala (L)	350	160	8.23	<0.001
	Nasal tip (C)	350	430	12.9	<0.001
Frontal region (upper) (C)	Zygomatic region (L)	450	418	−5.77	<0.001
	Oral commissure region (L)	450	390	−8.61	<0.001
	Mandibular angle region (L)	450	126	−7.8	<0.001
	Infraorbital region (L)	450	440	−3.65	0.015
	Temporal region (L)	450	390	−13.47	<0.001
	Lower eyelid (L)	450	430	−24.26	<0.001
	Upper eyelid (L)	450	430	−28.11	<0.001
	Lateral nasal surface (L)	450	50	−4.77	<0.001
	Philtrum (C)	450	388	6.67	<0.001
	Nasal ala (L)	450	160	8.59	<0.001
	Nasal tip (C)	450	430	13.84	<0.001
Frontal region (superolateral) (L)	Zygomatic region (L)	360	418	−5.76	<0.001
	Oral commissure region (L)	360	390	−8.45	<0.001
	Mandibular angle region (L)	360	126	−7.8	<0.001
	Infraorbital region (L)	360	440	−3.75	0.011
	Temporal region (L)	360	390	−13.05	<0.001
	Lower eyelid (L)	360	430	−23.21	<0.001
	Upper eyelid (L)	360	430	−26.84	<0.001
	Lateral nasal surface (L)	360	50	−4.86	<0.001
	Philtrum (C)	360	388	6.01	<0.001
	Nasal ala (L)	360	160	8.09	<0.001
	Nasal tip (C)	360	430	12.76	<0.001
Frontal region (C)	Zygomatic region (L)	360	418	−5.17	<0.001
	Oral commissure region (L)	360	390	−7.88	<0.001
	Mandibular angle region (L)	360	126	−7.4	<0.001
	Temporal region (L)	360	390	−12.47	<0.001
	Lower eyelid (L)	360	430	−22.62	<0.001
	Upper eyelid (L)	360	430	−26.25	<0.001
	Lateral nasal surface (L)	360	50	−4.58	<0.001
	Philtrum (C)	360	388	6.59	<0.001
	Nasal ala (L)	360	160	8.54	<0.001
	Nasal tip (C)	360	430	13.35	<0.001
Zygomatic region (L)	Mandibular angle region (L)	418	126	−3.87	0.007
	Supraorbital region (L)	418	350	4.38	0.001
	Temporal region (L)	418	390	−7.67	<0.001
	Lower eyelid (L)	418	430	−18.12	<0.001
	Upper eyelid (L)	418	430	−21.89	<0.001
	Philtrum (C)	418	388	12.12	<0.001
	Nasal ala (L)	418	160	12.72	<0.001
	Nasal tip (C)	418	430	19.3	<0.001
Oral commissure region (L)	Supraorbital region (L)	390	350	7.08	<0.001
	Infraorbital region (L)	390	440	5.05	<0.001
	Buccal region (L)	390	390	5.54	<0.001
	Temporal region (L)	390	390	−4.69	<0.001
	Lower eyelid (L)	390	430	−14.88	<0.001
	Upper eyelid (L)	390	430	−18.59	<0.001
	Philtrum (C)	390	388	14.75	<0.001
	Nasal ala (L)	390	160	14.77	<0.001
	Nasal tip (C)	390	430	21.87	<0.001
Mandibular angle region (L)	Supraorbital region (L)	126	350	6.85	<0.001
	Infraorbital region (L)	126	440	5.35	<0.001
	Buccal region (L)	126	390	5.72	<0.001
	Lower eyelid (L)	126	430	−8.4	<0.001
	Upper eyelid (L)	126	430	−10.96	<0.001
	Philtrum (C)	126	388	12.17	<0.001
	Nasal ala (L)	126	160	13.24	<0.001
	Nasal tip (C)	126	430	16.97	<0.001
Supraorbital region (L)	Temporal region (L)	350	390	−11.64	<0.001
	Lower eyelid (L)	350	430	−21.7	<0.001
	Upper eyelid (L)	350	430	−25.3	<0.001
	Lateral nasal surface (L)	350	50	−4.22	0.002
	Philtrum (C)	350	388	7.28	<0.001
	Nasal ala (L)	350	160	9.07	<0.001
	Nasal tip (C)	350	430	14	<0.001
Infraorbital region (L)	Temporal region (L)	440	390	−9.88	<0.001
	Lower eyelid (L)	440	430	−20.52	<0.001
	Upper eyelid (L)	440	430	−24.34	<0.001
	Philtrum (C)	440	388	10.15	<0.001
	Nasal ala (L)	440	160	11.22	<0.001
	Nasal tip (C)	440	430	17.38	<0.001
Buccal region (L)	Temporal region (L)	390	390	−10.23	<0.001
	Lower eyelid (L)	390	430	−20.55	<0.001
	Upper eyelid (L)	390	430	−24.26	<0.001
	Lateral nasal surface (L)	390	50	−3.41	0.035
	Philtrum (C)	390	388	9.22	<0.001
	Nasal ala (L)	390	160	10.54	<0.001
	Nasal tip (C)	390	430	16.2	<0.001
Temporal region (L)	Lower eyelid (L)	390	430	−10.08	<0.001
	Upper eyelid (L)	390	430	−13.79	<0.001
	Philtrum (C)	390	388	19.44	<0.001
	Nasal ala (L)	390	160	18.35	<0.001
	Nasal tip (C)	390	430	26.68	<0.001
Lower eyelid (L)	Upper eyelid (L)	430	430	−3.8	0.009
	Lateral nasal surface (L)	430	50	6.19	<0.001
	Philtrum (C)	430	388	29.97	<0.001
	Nasal ala (L)	430	160	26.21	<0.001
	Nasal tip (C)	430	430	37.68	<0.001
Upper eyelid (L)	Nasal sidewall (L)	430	50	7.92	<0.001
	Philtrum (C)	430	388	33.67	<0.001
	Nasal ala (L)	430	160	29.01	<0.001
	Nasal tip (C)	430	430	41.49	<0.001
Nasal sidewall (L)	Philtrum (C)	50	388	7.81	<0.001
	Nasal ala (L)	50	160	9.27	<0.001
	Nasal tip (C)	50	430	11.01	<0.001
Philtral ridge (C)	Nasal ala (L)	388	160	3.5	0.027
	Nasal tip (C)	388	430	6.73	<0.001

**Table 3:** Descriptive statistics (median and IQR) for subgroups defined by age and location.

Location	Age	Sample Size	Median	IQR (Interquartile Range)
Tear trough (L)	0–33	100	1192	391.75
	33–40	100	1294	201
	40–54	100	1073.5	287.75
	54–73	90	1304	341
Nasolabial fold (upper) (L)	0–33	100	1781	492.5
	33–40	100	1445.5	558.5
	40–54	100	1820.5	473.5
	54–73	90	1534.5	603
Upper lip vermilion (C)	0–33	120	1093	260.75
	33–40	100	1033.5	343.25
	40–54	100	1072	218.5
	54–73	70	1026	257.25
Glabella (C)	0–33	110	1559.5	522.25
	33–40	90	1599.5	266.5
	40–54	80	1199.5	592.25
	54–73	80	1457	436.75
Nasal dorsum (C)	0–33	30	1255	232.25
	33–40	18	1056	1272
	40–54	40	1289	223
	54–73	30	1411	240.5
Mental region (C)	0–33	120	1609.5	692.25
	33–40	100	1571	595.75
	40–54	110	1674.5	295.75
	54–73	100	1605	508.25
Lateral forehead (L)	0–33	110	1549	514.75
	33–40	90	1617.5	273
	40–54	70	1114	535
	54–73	80	1441	518
Upper forehead region (C)	0–33	130	1517	464.75
	33–40	100	1593	262.5
	40–54	120	1284.5	445.5
	54–73	100	1538	452.25
Superolateral forehead region (L)	0–33	110	1562.5	518
	33–40	90	1600	201.25
	40–54	70	1107	543.25
	54–73	90	1449	503.75
Frontal region (C)	0–33	110	1509.5	520.75
	33–40	90	1600.5	242.25
	40–54	80	1187.5	526
	54–73	80	1443	522.75
Zygomatic region (L)	0–33	110	1287.5	171
	33–40	100	1285.5	229.75
	40–54	118	1354.5	219
	54–73	90	1307	236.25
Oral commissure region (L)	0–33	100	1244.5	204
	33–40	100	1213.5	205.5
	40–54	110	1240	244
	54–73	80	1229.5	367.75
Mandibular angle region (L)	0–33	48	1244.5	239.75
	40–54	48	1186	182.75
	54–73	30	1174.5	165.25
Supraorbital region (L)	0–33	110	1498.5	491.25
	33–40	90	1556.5	277.5
	40–54	70	1111.5	478.25
	54–73	80	1441.5	499.25
Infraorbital region (L)	0–33	130	1398	320.25
	33–40	100	1379	383.25
	40–54	110	1365	196
	54–73	100	1300	269.75
Buccal region (L)	0–33	120	1389.5	355
	33–40	100	1365	523.75
	40–54	80	1372	250.5
	54–73	90	1309.5	384.5
Temporal region (L)	0–33	100	998.5	169.75
	33–40	100	1175.5	266.5
	40–54	100	1151	188.75
	54–73	90	1199.5	223.25
Lower eyelid (L)	0–33	120	802.5	233.25
	33–40	100	780	196.25
	40–54	110	814.5	231.25
	54–73	100	885.5	272
Upper eyelid (L)	0–33	120	569	140
	33–40	100	575	136
	40–54	110	562	105.5
	54–73	100	597	143.5
Lateral nasal surface (L)	0–33	10	981.5	264
	33–40	30	1197	868.75
	54–73	10	981.5	264
Philtrum (C)	0–33	110	1464	276.5
	33–40	88	1710.5	308.5
	40–54	90	1607	287.25
	54–73	100	1729.5	393.5
Nasal ala (L)	0–33	40	1751	192.25
	33–40	40	1584	550.75
	40–54	40	1790.5	123.75
	54–73	40	1750	202.5
Nasal tip (C)	0–33	120	1632	327.5
	33–40	100	1969.5	270.5
	40–54	110	2013	459.25
	54–73	100	2045	360

## Data Availability

The original contributions presented in this study are included in the article. Further inquiries can be directed to the corresponding author.
